# HIBCH mutations can cause Leigh-like disease with combined deficiency of multiple mitochondrial respiratory chain enzymes and pyruvate dehydrogenase

**DOI:** 10.1186/1750-1172-8-188

**Published:** 2013-12-04

**Authors:** Sacha Ferdinandusse, Hans R Waterham, Simon JR Heales, Garry K Brown, Iain P Hargreaves, Jan-Willem Taanman, Roxana Gunny, Lara Abulhoul, Ronald JA Wanders, Peter T Clayton, James V Leonard, Shamima Rahman

**Affiliations:** 1 Academic Medical Centre, Laboratory Genetic Metabolic Diseases, University of Amsterdam, Amsterdam, The Netherlands; 2 Chemical Pathology, Great Ormond Street Hospital, London, UK; 3 Diagnostic Imaging, Great Ormond Street Hospital, London, UK; 4 Metabolic Unit, Great Ormond Street Hospital, London, UK; 5 Neurometabolic Unit, National Hospital for Neurology, London, UK; 6 Mitochondrial Research Group, Clinical and Molecular Genetics Unit, UCL Institute of Child Health, 30 Guilford Street, London WC1N 1EH, UK; 7 Department of Biochemistry, University of Oxford, Oxford, UK; 8 Department of Clinical Neurosciences, UCL Institute of Neurology, London, UK

**Keywords:** Mitochondrial disease, Multiple respiratory chain enzyme deficiencies, Pyruvate dehydrogenase deficiency, 3-hydroxy-isobutyryl-CoA hydrolase, HIBCH, Acylcarnitines, Multiple mitochondrial dysfunctions syndrome, Valine catabolism, Organic aciduria

## Abstract

**Background:**

Deficiency of 3-hydroxy-isobutyryl-CoA hydrolase (HIBCH) caused by *HIBCH* mutations is a rare cerebral organic aciduria caused by disturbance of valine catabolism. Multiple mitochondrial respiratory chain (RC) enzyme deficiencies can arise from a number of mechanisms, including defective maintenance or expression of mitochondrial DNA. Impaired biosynthesis of iron-sulphur clusters and lipoic acid can lead to pyruvate dehydrogenase complex (PDHc) deficiency in addition to multiple RC deficiencies, known as the multiple mitochondrial dysfunctions syndrome.

**Methods:**

Two brothers born to distantly related Pakistani parents presenting in early infancy with a progressive neurodegenerative disorder, associated with basal ganglia changes on brain magnetic resonance imaging, were investigated for suspected Leigh-like mitochondrial disease. The index case had deficiencies of multiple RC enzymes and PDHc in skeletal muscle and fibroblasts respectively, but these were normal in his younger brother. The observation of persistently elevated hydroxy-C4-carnitine levels in the younger brother led to suspicion of HIBCH deficiency, which was investigated by biochemical assay in cultured skin fibroblasts and molecular genetic analysis.

**Results:**

Specific spectrophotometric enzyme assay revealed HIBCH activity to be below detectable limits in cultured skin fibroblasts from both brothers. Direct Sanger sequence analysis demonstrated a novel homozygous pathogenic missense mutation c.950G <A; p.Gly317Glu in the *HIBCH* gene, which segregated with infantile-onset neurodegeneration within the family.

**Conclusions:**

HIBCH deficiency, a disorder of valine catabolism, is a novel cause of the multiple mitochondrial dysfunctions syndrome, and should be considered in the differential diagnosis of patients presenting with multiple RC deficiencies and/or pyruvate dehydrogenase deficiency.

## Background

Mitochondrial disorders affect approximately 1 in 5000 births, and are clinically, biochemically and genetically heterogeneous [1]. Combined deficiency of multiple respiratory chain (RC) enzymes is one of the most frequent findings in children with suspected mitochondrial disease, representing approximately 30% of cases in whom a biochemical abnormality is identified. Approximately 50% of patients with multiple RC deficiencies have impaired replication or maintenance of the mitochondrial DNA (mtDNA), leading to progressive depletion of mtDNA [2] or accumulation of multiple mtDNA deletions. The remaining ~50% of cases have heterogeneous underlying causes, including mitochondrial or nuclear-encoded defects of mitochondrial protein synthesis [3] and the multiple mitochondrial dysfunctions syndrome, in which the activity of PDHc is also impaired [4-6]. Defects in mtDNA repair, maintenance or translation result in combined deficiency of complexes I, III and IV (i.e. complexes that contain mtDNA-encoded subunits) whereas the multiple mitochondrial dysfunctions syndrome usually affects complexes containing iron-sulphur (Fe-S) clusters (complexes I, II and III) as well as PDHc.

**Figure 1:**
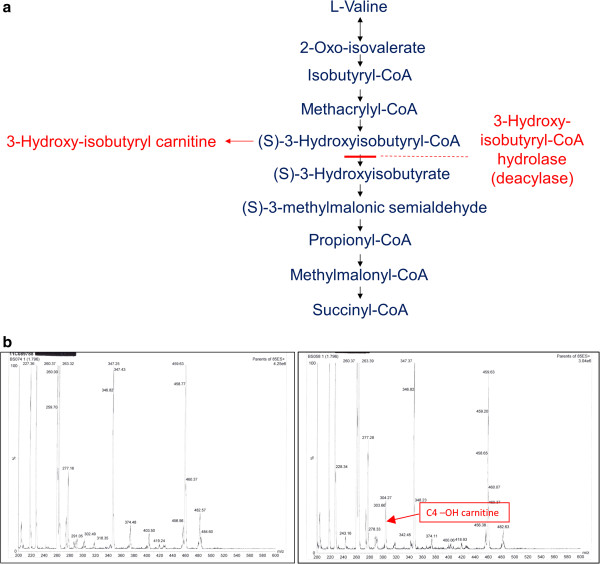
**HIBCH deficiency leads to accumulation of hydroxy-C4-carnitine. (a)** Valine degradation pathway. 3-Hydroxy-isobutyrylCoA hydrolase (HIBCH) catalyses the fifth step of valine catabolism. HIBCH deficiency leads to accumulation of 3-hydroxy-isobutyryl carnitine, which is detected as hydroxy-C4-carnitine by tandem mass spectrometry. **(b)** Plasma acylcarnitine analysis by tandem mass spectrometry. Left panel: normal acylcarnitine profile; Right panel: acylcarnitine profile from Patient 2 with accumulating hydroxy-C4-carnitine indicated by arrow.

Neurological features of cerebral organic acidurias (disorders of degradation of the carbon skeleton of amino acids) can be clinically and radiologically indistinguishable from mitochondrial encephalomyopathies caused by primary RC deficiencies; seizures, neurological regression and bilateral symmetrical basal ganglia lesions may occur in both groups of disorders [7-10]. 3-Hydroxy-isobutyryl-CoA hydrolase (HIBCH) is a mitochondrial enzyme that catalyses the fifth step of valine catabolism, the conversion of 3-hydroxy-isobutyryl-CoA to 3-hydroxy-isobutyrate (Figure 1a). HIBCH deficiency has previously been reported in only two patients [11,12]. We now describe two new genetically confirmed cases (siblings), one of whom presented with combined defects of multiple RC enzymes and the pyruvate dehydrogenase complex (PDHc). This potentially represents a new disease mechanism mimicking the multiple mitochondrial dysfunctions syndrome, namely degradation of multiple enzymes resulting from accumulation of a toxic metabolite methacrylyl-CoA that is postulated to reduce mitochondrial enzyme activities by reacting with exposed thiol groups.

## Methods

### Patient 1

The index case was the first child of healthy distantly related Pakistani parents. He was born at term weighing 3.2 kg. There were no neonatal problems, but from 3 months he had developmental regression, with loss of smile and progressive hypotonia. At 8 months Nissen fundoplication was performed because of persistent vomiting. From 8 months he developed myoclonic jerks and from 10 months recurrent generalised seizures. Cerebrospinal fluid (CSF) lactate was mildly elevated at 2.2 mmol/l (reference range <2 mmol/l) at 8 months, with a normal venous blood lactate of 1.7 mmol/l (reference range < 1.8 mmol/l). A repeat CSF lactate was 3.5 mmol/l. Histological examination of an open muscle biopsy performed at 17 months revealed a mild increase in neutral lipid droplets, but no ragged-red or cytochrome oxidase (COX)-negative fibres. His clinical course was marked by persistent vomiting and retching, despite the fundoplication, and extreme irritability and sleep disturbance. MRI brain showed altered signal and atrophy in the globi pallidi and relative sparing of the thalami. Leukoencephalopathy and some generalised atrophy were also observed. He died at 3 years of age.

### Patient 2

**Figure 2:**
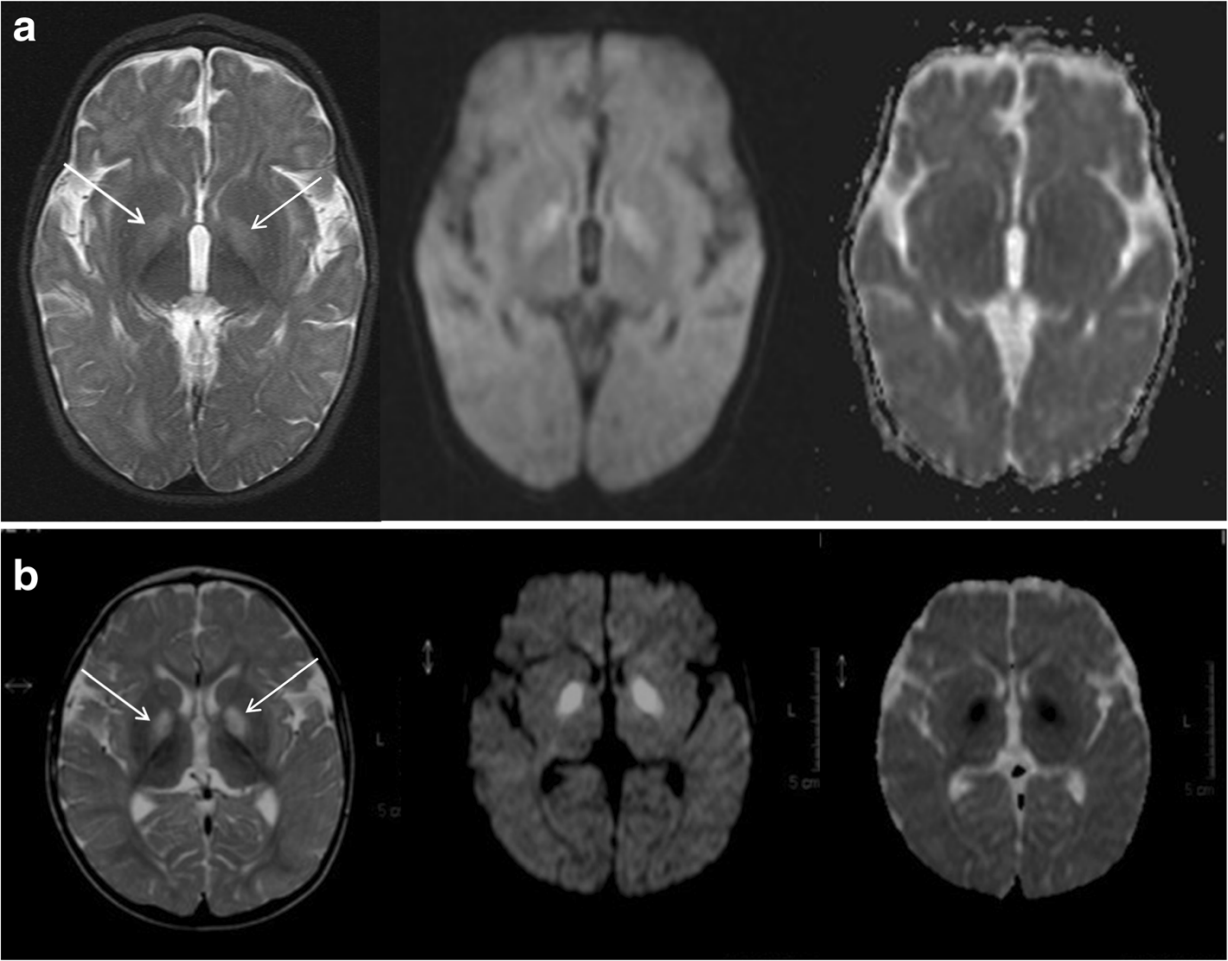
**Magnetic resonance imaging of the brain in HIBCH deficiency. (a)** Patient 2 at 9 months: axial T2 weighted image (left panel) shows bilateral symmetrical signal hyperintensity within the globi pallidi (arrows) accompanied by signal hyperintensity on diffusion weighted image (middle panel) and low signal on ADC map (right panel) in keeping with restricted diffusion which is beginning to pseudonormalise on ADC map. The features are those of a subacute neurometabolic insult. In addition there is some generalised non-specific lack of cerebral volume with prominence of the cerebral sulci and delay in myelin maturation. **(b)** Patient 3 at 11 months: axial T2 (far left) shows bilateral symmetrical signal hyperintensity and swelling in the globi pallidi (arrows) with restricted diffusion (diffusion weighted image middle panel, ADC map right panel) consistent with cytotoxic oedema. The imaging pattern is suggestive of an acute neurometabolic insult.

A younger sister of Patient 1 is currently well at 9 years of age but the third child in the family, Patient 2, was a boy born at term after a normal pregnancy, with a birth weight of 2.9 kg. He presented on day 1 of life with poor sucking and feeding difficulties. Feeding problems continued and were associated with poor weight gain, necessitating nasogastric tube feeding from 2 months. Echocardiogram and ophthalmological examination were normal at 4 months. He subsequently developed infantile spasms, and hypsarrhythmia was observed on the electroencephalogram (EEG) at 7 months. The infantile spasms responded dramatically to high dose prednisolone therapy. He also had a progressive dystonic disorder and persistent vomiting despite fundoplication (performed at 10 months), associated with abdominal pain, extreme irritability, sleep disturbance and breath-holding episodes leading to severe oxygen desaturation. Venous blood lactate was elevated at 4 mmol/l on one occasion but normal at other times (Table 1). CSF lactate was mildly elevated on two occasions at 2.1 and 2.6 mmol/l. The most significant biochemical abnormality was persistent elevation of hydroxy-C4-carnitine (0.77 – 1.25 μmol/L, reference range <0.4; Figure 1b), an investigation which was not available when Patient 1 was alive. Normal biochemical investigations included very long chain fatty acids, transferrin electrophoresis, purines, creatine kinase, cholesterol and triglycerides, CSF neurotransmitters, urinary glycosaminoglycans and white cell enzyme assays for lysosomal storage disorders. Muscle biopsy showed mildly increased lipid deposition within muscle fibres but no ragged-red or COX-negative fibres. MRI brain at 11 months showed abnormal signal within the dentate nuclei and the globi pallidi, with a generalised lack of white matter (see Figure 2a and legend for details of imaging appearances). Relentless progression led to death at 2 years 8 months.

**Table 1:** Clinical and biochemical features of HIBCH deficiency

	**Patient 1**	**Patient 2**	**Patient 3 (previously reported in Loupatty et al. 2007 **[12]**)**	**Patient reported by Brown et al. 1982**[11]
Gender	Male	Male	Male	Male
Age at presentation	3 months	Birth	4 months	Birth
Initial presentation	Developmental regression	Poor feeding	Head bobbing	Dysmorphic features
Age at death	3 years	2 years 8 months	Alive at 8 years	3 months
Family history	Distantly related British Pakistani parents	Distantly related parents; younger sibling of Patient 1	Unrelated parents	First cousin Egyptian parents
Neonatal problems	Vomiting	Poor feeding	Poor feeding	Poor feeding
Hypotonia	++	++	+	++
Dystonia	+	++	++	NS
Seizures	Myoclonus from 8 months; generalised seizures from 10 months	Infantile spasms	From 9 months - transient absences and episodes of eye rolling	NS
Developmental regression	+	+	+	NS
Episodes of acute encephalopathy	No	No	+	NS
Other clinical features	Screaming episodes, sleep disturbance, central apnoea, vision impairment (optic atrophy), hearing loss, microcephaly	Recurrent episodes of screaming, breath-holding, poor sleep, central apnoea, visual im-pairment, microcephaly	Cerebellar ataxia - truncal ataxia, dysmetria and intention tremor	Facial dysmorphism, tetralogy of Fallot, multiple vertebral anomalies, agenesis of cingulate gyrus and corpus callosum (PM findings)
MRI brain	Altered signal and atrophy in the globi pallidi, with leukoencephalo-pathy and some generalised atrophy	Abnormal signal within the dentate nuclei and the globi pallidi, with a generalised lack of white matter (Figure 2a)	Signal abnormalities in globi pallidi and midbrain and asymmetric involvement of cerebral peduncles (Figure 2b)	ND
Venous blood lactate (<2.0 mmol/L)	1.7	**4.0**, 1.6, 1.2	1.7	NS
CSF lactate (<2.0 mmol/L)	**3.5, 2.2**	**2.1, 2.6**	1.3	NS
Hydroxy-C4-carnitine (<0.4 µmol/L)	ND	**0.77-1.25**	**0.45-1.73**	ND
**Muscle respiratory chain enzyme activities (ratio to citrate synthase activity)**				
Complex I (0.104-0.268)	**0.068**	0.211	**0.089**	ND
Complexes II + III (0.040-0.204)	**0.010**	0.056	0.096	ND
Complex IV (COX) (0.014-0.034)	**0.010**	0.016	**0.013**	ND
**Muscle glutathione levels**				
Muscle GSH (8.5-16.7 μmol/mg)	ND	ND	**7.9**	ND
**Muscle mtDNA levels determined by Southern blot analysis**				
Muscle mtDNA (arbitrary units relative to the multicopy nuclear 18S rRNA gene) Paediatric controls (n = 7): Mean 17.3, SD 5.3, range 12.2 - 27.8	ND	ND	29.5	ND
**Fibroblast enzyme activities**				
PDHc (0.7-1.1 nmol/(min.mg))	**0.3**	0.73	**0.62**	ND
COX (30–90 nmol/(min.mg))	**25**	ND	117	ND
HIBCH (7.9 ± 1.3 nmol/(min.mg))	**<2.6**	**<2.6**	**<2.6**	**~20%** of controls
***HIBCH*** **mutations**	Homozygous c.950G <A; p.G317E	Homozygous c.950G <A; p.G317E	Compound heterozygous c.365A <G; p.Y122C and IVS2-3C <G; p.R27fsX50	Homozygous c.219_220insTTGAATAG; p.K73fsX86

Key: COX = cytochrome oxidase; GSH = glutathione; ND = not determined; NS = not stated in original report; PDHc = pyruvate dehydrogenase complex; PM = post mortem; numbers in bold are outside the reference range, indicated in the left column.

The family history is notable in that a maternal uncle of Patients 1 and 2 (whose own parents were first cousins) had a progressive neurological disorder from the age of 14 years, eventually leading to death at 46 years. His sister, the maternal aunt of Patients 1 and 2, had a neuromuscular condition from birth, but normal cognition, and died at 47 years.

### Patient 3

Patient 3 is the first child of healthy unrelated white European parents. He presented at 4 months with developmental delay and ataxia and subsequent neurological regression, and his case history has been reported previously [12]. We now present additional biochemical data from this case, including muscle glutathione levels, and compare his neuro-imaging features to those of Patient 2 (Figure 2b).

In all three cases, the following diagnostic investigations were performed after informed parental consent. Clinical and biochemical details of these 3 patients, and a fourth patient previously reported in the literature (Brown et al. [11]) are summarised in Table 1.

### Biochemical analyses

Previously reported spectrophotometric assay methods were used to determine the activities of RC enzyme complexes I, II + III and IV in frozen skeletal muscle homogenates [13] and complex IV in cultured skin fibroblasts [14]. Quantitative immunocytochemistry was used to investigate relative expression of complex IV subunit 1 (MTCO1) in patient and control fibroblasts, which were cultured in the presence of 5 μM MitoTracker® Red CM-H2-XRos (Molecular Probes Inc., Oregon) to label mitochondria red, followed by green immunostaining with an anti-MTCO1 monoclonal antibody (Clone 1D6E1A8, Abcam) as described [15]. Densitometric analysis of images captured using a Zeiss Axiophot epifluorescence microscope was performed as reported previously [15] and statistical analysis was performed using Student’s *t* test. PDHc activity was measured in cultured skin fibroblasts using a radiochemical method [16]. HIBCH activity was determined by spectrophotometric enzymatic assay using the physiological substrate S-3-hydroxyisobutyryl-CoA, as previously described [12]. Glutathione in frozen skeletal muscle homogenates was assayed by electrochemical high performance liquid chromatography using a previously reported method [17].

### Molecular genetic analysis

Genomic DNA was extracted from cultured skin fibroblasts using standard methods. Exons and flanking intronic sequences of the *HIBCH* gene were sequenced after amplification by PCR from genomic DNA using intronic primers with -21 M13 (5'-TGTAAAACGACGGCCAGT-3') or M13-Rev (5'-CAGGAAACAGCTATGACC-3') extensions (Additional file 1: Table S1). PCR fragments were sequenced with -21 M13 and M13-Rev sequence primers using BigDye Terminator cycle sequencing kits (Applied Biosystems, Foster City, CA, USA). Sequence data were compared to the reference *HIBCH* sequence (GenBank accession number, NM_014362.2) with nucleotide numbering starting at the first adenine of the translation initiation codon ATG.

All diagnostic investigations were performed after obtaining informed parental consent. This study was approved by the National Research Ethics Committee London Bloomsbury, UK.

## Results

### Biochemical analyses

**Figure 3:**
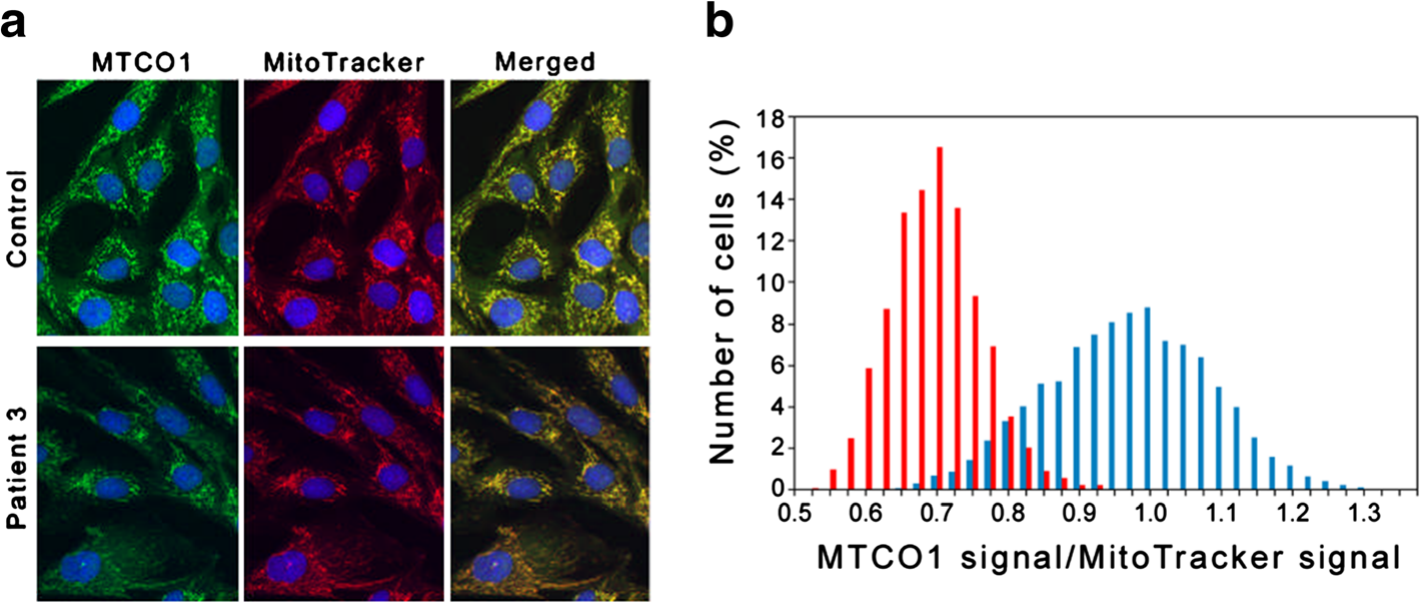
**Cytochrome oxidase immunocytochemistry in cultured skin fibroblasts.** Expression of complex IV subunit 1 (MTCO1) in control and Patient 3 fibroblasts. Cells were cultured in the presence of 5 μM MitoTracker® Red CM-H2-XRos to label mitochondria red, followed by green immunostaining with anti-MTCO1 antibodies and blue nuclear counterstaining with DAPI. **(a)** Pseudo-coloured fluorescent micrographs. **(b)** Relative cellular expression levels of MTCO1 in Control (blue bars) and Patient (red bars) cells. The mean grey levels were measured for both the green and red images of 3631 control and 2184 patient cells, and the ratio of the two signals was calculated for each cell. The histogram reveals the frequency of cells with a particular MTCO1/MitoTracker ratio at intervals of 0.025. The total number of cells per culture is 100%.

Patient 1 had combined mitochondrial enzyme defects involving PDHc and multiple RC enzymes: residual enzyme activities in skeletal muscle were 65%, 25% and 71% of the lowest control for complexes I, II + III and IV respectively, whilst fibroblast PDHc activity was 43% of the lowest control (Table 1). RC and PDHc activities were normal in Patient 2, whilst Patient 3 had mild reduction of complexes I and IV in skeletal muscle (Table 1). Quantitative immunocytochemistry confirmed significantly decreased expression of complex IV subunit I in cultured skin fibroblasts from Patient 3 (Figure 3); mean cellular MTCOI expression in Patient 3 fibroblasts was 66% compared to controls (p < 0.001). The presence of persistently elevated plasma hydroxy-C4-carnitine in Patients 2 and 3 suggested the possibility of HIBCH deficiency, which was confirmed by enzyme assay; fibroblast HIBCH activity was below detectable limits in all three patients (Table 1).

### Molecular genetic analysis

**Figure 4:**
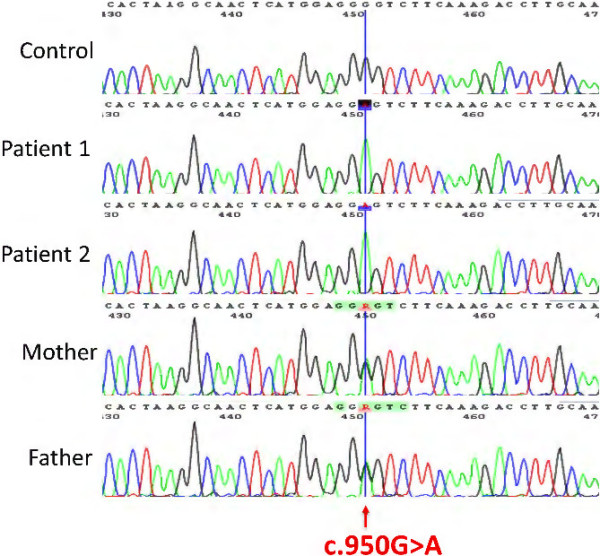
**Homozygous *HIBCH *mutation in patients 1 and 2.** Sequence electropherograms of *HIBCH* gene. Top panel: Control; Second (from top) panel: Patient 1; Third panel: Patient 2; Fourth panel: Mother; Fifth panel: Father. Both patients are homozygous for the c.950G <A mutation, whilst the parents are heterozygous.

Sequence analysis revealed a novel homozygous missense mutation c.950G <A;p.Gly317Glu in the *HIBCH* gene on chromosome 2q32.3 in both Patients 1 and 2 (Figure 4). The mutation is absent in the dbSNP and 1000 genomes databases (which includes 200 Pakistani alleles), affects an amino acid residue highly conserved among different species and is predicted by the SIFT software as deleterious and by the Polyphen-2 software as probably damaging. Parents and the maternal aunt and uncle were all heterozygous for the c.950G <A mutation. Patients 1 and 2 were also homozygous for two known single nucleotide polymorphisms within the *HIBCH* gene: c.2T <C;p.Met1? (disruption main translation initiation codon; initiation 5 codons downstream). and c.136A <G;p.Thr46Ala. The reported Minor Allele Frequencies for the two polymorphisms are for c.2T <C: T = 0.3988 (European American) and C = 0.4557 (African American); and for c.136A <G: A = 0.2407 (European American) and A = 0.3443 (African American).

Molecular analysis of Patient 3 has previously been reported [12].

## Discussion

Two brothers with a progressive neurodegenerative disorder are reported. Patient 1 had combined mitochondrial enzyme defects involving PDHc and multiple RC enzymes (complexes I, II + III and IV, Table 1) whereas these were normal in Patient 2. However, the remarkably similar clinical features in both brothers implied that they had the same underlying condition. The presence of persistently elevated plasma hydroxy-C4-carnitine in Patient 2 (Figure 1b, Table 1) suggested the possibility of HIBCH deficiency, which was confirmed by enzyme assay in cultured skin fibroblasts and molecular genetic analysis. Fibroblast HIBCH activity was below detectable limits in both patients (Table 1), and sequence analysis revealed a homozygous missense mutation c.950G <A (p.Gly317Glu) in the *HIBCH* gene (Figure 4). The causal nature of this mutation is indicated by the fact that this mutation 1) concerns a highly conserved amino acid; 2) is predicted *in silico* to be deleterious and probably damaging; 3) is the only putative mutation in the coding region of the *HIBCH* gene encoding HIBCH, an enzyme which was fully deficient in the patients; and 4) has not been observed previously in genome databases, including 200 ethnically matched control alleles. Finally, the mutation segregated with the Leigh-like disease in this family and was heterozygous in the unaffected parents and the maternal aunt and uncle who had a different neurological phenotype. Although we performed extensive metabolic investigations in Patient 1 which did not reveal any other metabolic disturbances, we cannot completely exclude the small possibility that a second autosomal recessive disorder may have been present, which could have accounted for the more severe biochemical defects observed in this patient.

HIBCH deficiency has been reported in only two cases previously including Patient 3 [11,12]. Table 1 summarises the clinical and biochemical findings in all 4 cases. Consistent features of this condition appear to be hypotonia, poor feeding and developmental regression with seizures starting in infancy (Table 1) and symmetrical involvement of the globi pallidi seen on brain MRI. Images were no longer available from Patient 1 for review, but acute changes suggestive of cytotoxic oedema, shown by imaging changes of restricted diffusion with subsequent evolution to mature scarring, were seen in Patient 2 and also in Patient 3 (previously reported in [12]). The subthalamic regions were also involved in Patient 3. The predilection for the basal ganglia in HIBCH deficiency is intriguing, and the pathomechanism(s) underlying the basal ganglia lesions, and in particular the selective involvement of the globi pallidi and subthalamic nuclei, are not clear. It is possible that the lesions arise from localised cerebral energy failure and subsequent neuronal cell death, as in other mitochondrial diseases including Leigh syndrome (subacute necrotizing encephalomyelopathy) and Kearns-Sayre syndrome [7,8]. Alternatively, the underlying pathological mechanisms and the imaging changes may more closely resemble those seen in other organic acidurias, such as glutaric aciduria type I and methylmalonic and propionic acidurias, which remain poorly understood [9,10]. However one hypothesis suggests that one mechanism involves reaction of acrylyl-CoA and glutaconyl-CoA with thiol groups [18] and glutathione depletion has been noted in methylmalonic acidaemia (MMA) [19]. It is also interesting to note that there is some evidence for secondary RC defects as a possible mechanism of basal ganglia damage in these organic acidaemias [20,21], and one study demonstrated competitive inhibition of PDHc by propionyl-CoA, as well as inhibition of complex III and the α-ketoglutarate dehydrogenase complex [22].

Most cases of multiple RC deficiencies reported in the literature are caused by defects of maintenance or translation of the mtDNA. The former group includes defects of nuclear genes involved in replication of the mtDNA and defects of nucleoside salvage necessary to provide dNTP building blocks for mtDNA synthesis [23]. In the previously reported Patient 3, mtDNA copy number was normal in skeletal muscle (Table 1) and cultured myoblasts and skin fibroblasts (data not shown), excluding the possibility of mtDNA depletion as the cause of the mild RC defects observed in this patient. Disorders of mitochondrial translation may be primary mtDNA defects, such as deletions of mtDNA or point mutations involving the mitochondrial transfer RNA genes, or nuclear gene defects [3]. In a small number of cases, a combined deficiency of multiple RC enzymes associated with PDHc deficiency has been reported, the so-called multiple mitochondrial dysfunctions syndrome. Recently, mutations in two Fe-S cluster assembly proteins were shown to cause multiple mitochondrial enzyme defects including PDHc deficiency. Mutations in NFU1, required late in the maturation of Fe-S clusters, caused a syndrome of fatal infantile encephalopathy and/or pulmonary hypertension in Spanish and Mexican families with combined defects of PDHc, complex II, lipoic acid synthase and the glycine cleavage system [5,6]. Patients with mutations in *BOLA3*, which is also needed for Fe-S and lipoic acid biosynthesis, have a similar biochemical phenotype [6,24], but the precise pattern of RC deficiencies in these defects differs according to the specific Fe-S cluster synthesis pathway that is impaired [6].

With respect to the pathological mechanism underlying HIBCH deficiency, methacrylyl-CoA, a proximal metabolite in the valine degradation pathway (Figure 1a), could well play a causative role. Cysteine and cysteamine conjugates of methacrylyl-CoA were shown to accumulate in multiple tissues, particularly liver, kidney and brain, in the first patient reported with HIBCH deficiency [11]. Cysteine is a non-essential amino acid bearing a thiol side chain that is frequently involved in enzymatic reactions and is susceptible to oxidation, leading to formation of the disulphide cystine. Cystine plays an important structural role in many proteins; disulphide bonds are important in crosslinking proteins, serving to increase rigidity and conferring resistance to proteolytic degradation. Methacrylyl-CoA could react with mitochondrial enzymes containing essential cysteine residues, including PDHc and RC enzymes [25-29], thereby reducing their activities. Depletion of cysteine also could lead to reduced activity of mitochondrial enzymes containing Fe-S clusters (e.g. RC complexes I, II and III and the Krebs cycle enzyme aconitase), since cysteine provides sulphide for Fe-S clusters. The ability of thiol groups to undergo redox reactions confers antioxidant properties to cysteine, most importantly in the tripeptide glutathione (composed of cysteine, glycine and glutamic acid) and also in mitochondrial thioredoxin. Thioredoxin contains two cysteine residues in its active centre that undergo reversible oxidation/reduction in response to mitochondrial redox status [30]. Depletion of mitochondrial pools of cysteine, glutathione and thioredoxin by methacrylyl-CoA could lead to oxidative damage, with further impairment of RC enzyme function. Indeed, we found mildly reduced muscle glutathione in Patient 3, who also had mildly reduced activities of RC complexes I and IV in muscle (Table 1), and significantly decreased expression of complex IV subunit I in fibroblasts measured by quantitative immunocytochemistry (Figure 3). These effects are likely to vary according to the levels of oxidative stress, which may explain the variable RC and PDHc defects seen in the 3 patients with HIBCH deficiency (Table 1). We have previously demonstrated reversibility of RC defects in Patient 3, who had normal activities of complexes I-IV in a biopsy performed 3 months after the abnormal biopsy shown in Table 1 with mildly reduced complex I and IV levels (Loupatty et al. 2007 [12]). A reduced plasma concentration of glutathione has been found in both mitochondrial disorders and organic acidaemias [31,32]. Complex I dysfunction was linked to Parkinson’s disease (PD) following the observation of a high incidence of PD in subjects who had recreationally misused 1-methyl-4-phenyl-1,2,3,6-tetrahydropyridine (MPTP), a known inhibitor of complex I [33] and glutathione depletion has been noted to precede complex I deficiency in PD [34]. Increased oxidation of six complex I cysteine residues was demonstrated in glutathione-depleted mouse brains, an oxidative stress model of murine PD [35]. Methacrylyl-CoA acid could further disrupt enzymatic reactions taking place in mitochondria by irreversibly binding cofactors such as CoA and lipoic acid. Lack of CoA would be expected to inhibit the Krebs cycle, whilst lipoate is an essential component of PDHc.

Toxic damage to mitochondrial enzymes has previously been demonstrated in ethylmalonic encephalopathy. In affected patients mutations of the ETHE1 sulphur dioxygenase lead to accumulation of sulphide, which affects the function of complex IV and the short chain acyl-CoA dehydrogenase involved in mitochondrial fatty acid oxidation [36,37]. By contrast, complex IV activity appears to be only mildly reduced in HIBCH deficiency, with relatively greater effects on complex I and PDHc observed in Patient 1 in this study.

These cases illustrate the clinical utility of acylcarnitine analysis in the differential diagnosis of suspected mitochondrial disease. The older brother Patient 1 had died before the introduction of blood acylcarnitine analysis into routine metabolic clinical practice, but the persistently elevated hydroxy-C4-carnitine levels in his younger brother Patient 2 eventually led to the correct diagnosis of HIBCH deficiency. Acylcarnitine analysis is a useful investigation in other cases of suspected mitochondrial disease, since it may help to direct diagnostic genetic investigations, for example succinyl and propionyl carnitines may be elevated in patients with succinyl-CoA ligase deficiency caused by *SUCLA2* or *SUCLG1* mutations [38,39].

## Conclusions

In conclusion, HIBCH deficiency should be considered in the differential diagnosis for patients with multiple RC defects, including those with associated PDHc deficiency. Moreover, the presence of hydroxy-C4-carnitine in the acylcarnitine profile should alert the clinician to the possibility of this condition. Our results stress the importance of acylcarnitine analysis in patients with suspected mitochondrial disease.

## Competing interests

The authors declare that they have no competing interests.

## Authors’ contributions

SF performed biochemical studies, interpreted data and revised manuscript. HRW performed molecular genetic studies, interpreted data and revised manuscript. SJRH, GB, IPH, J-WT, RG, LA, RJAW acquired and interpreted data. PTC and JVL interpreted data and revised manuscript. SR conceived study, acquired and interpreted data, and wrote manuscript. All authors read and approved the final manuscript.

## Supplementary Material

Additional file 1: Table S1 *HIBCH*-specific PCR primers used for sequence analysis of *HIBCH.* Click here for file
