# Supplementary material for: HIBCH mutations can cause Leigh-like disease with combined deficiency of multiple mitochondrial respiratory chain enzymes and pyruvate dehydrogenase
Source: Orphanet J Rare Dis. 2013 Dec 4;8:188. doi: 10.1186/1750-1172-8-188 (PMC4222069; doi:10.1186/1750-1172-8-188)
Supplement: Additional file 1: Table S1 — HIBCH-specific PCR primers used for sequence analysis of HIBCH. [file 1750-1172-8-188-S1.docx]

***HIBCH* mutations can cause Leigh-like disease with combined deficiency of multiple mitochondrial respiratory chain enzymes and pyruvate dehydrogenase**

Sacha Ferdinandusse, PhD^1^, Hans R Waterham, PhD^1^, Simon JR Heales, PhD^2,5,6^, Garry K Brown, PhD^7^, Iain P Hargreaves, PhD^5^, Jan-Willem Taanman, PhD^8^, Roxana Gunny, FRCR^3^, Lara Abulhoul, MRCPCH^4^, Ronald JA Wanders, PhD^1^, Peter T Clayton, FRCP MD^6^, James V Leonard, FRCP PhD^6^ and Shamima Rahman, FRCP PhD^4,6^*

^1^Academic Medical Centre, Laboratory Genetic Metabolic Diseases, University of Amsterdam, The Netherlands; ^2^Chemical Pathology, Great Ormond Street Hospital, London, UK; ^3^Diagnostic Imaging, Great Ormond Street Hospital, London, UK; and ^4^Metabolic Unit, Great Ormond Street Hospital, London, UK; ^5^Neurometabolic Unit, National Hospital for Neurology, London, UK; ^6^Clinical and Molecular Genetics Unit, UCL Institute of Child Health, London, UK; ^7^Department of Biochemistry, University of Oxford, Oxford, UK; ^8^ Department of Clinical Neurosciences, UCL Institute of Neurology, London, UK

* Author for correspondence

**Address for correspondence:**

Mitochondrial Research Group, Clinical and Molecular Genetics Unit, UCL Institute of Child Health, 30 Guilford Street, London WC1N 1EH, UK

Tel: +44(0)207 905 2608

Fax: +44(0)207 404 6191

Email: shamima.rahman@ucl.ac.uk

**Supplementary Table 1: *HIBCH*-specific PCR primers used for sequence analysis of *HIBCH* gene**

| **Primers** | **Sequence (5’→3’)^1^** |
| --- | --- |
| IN1-30HIBCHf  IN2+369HIBCHr | [-21M13]- ATTCTCGCTCTGCTGCTTTAG  [M13-Rev]-TAGCCTGAAAGTTCCAAATGC |
| IN2-234HIBCHf  IN3+152HIBCHr | [-21M13]-TCCAACCATTGAAGAACATC  [M13-Rev]-TCTTATGCCACCTCCTACAAG |
| IN3-161HIBCHf  IN4+189HIBCHr | [-21M13]-GCTTTGGAATTGTTCGTCTAC  [M13-Rev]-TCTGTCTCAGGAGGCAGTAAG |
| IN4-142HIBCHf  IN5+348HIBCHr | [-21M13]-GGATGAGATTCGTTCTTCTG  [M13-Rev]-AACATGGCCAGATAGTCAG |
| IN5-463HIBCHf  IN6+75HIBCHr | [-21M13]- TGTGATGATGGCTCACTGTAG  [M13-Rev]-GTAAGGATGCCTATTGATTGC |
| IN6-140HIBCHf  IN7+70HIBCHr | [-21M13]- CCATACATTTAGTCGTCTTTG  [M13-Rev]-CCACACAATTATGTTTCAATGAG |
| IN7-435HIBCHf  IN8+71HIBCHr | [-21M13]-CAGCTAGGCCAGGTGATATAG  [M13-Rev]-TCCATCAATTCCTTCAACAAC |
| IN8-173HIBCHf  IN9+108HIBCHr | [-21M13]- AATACCCAAGTTAGCACAGTTC  [M13-Rev]-ATAGGTCCAGGATTCACACC |
| IN9-343HIBCHf  IN10+136HIBCHr | [-21M13]- GTTGTGCATTGTCTGTGGTAG  [M13-Rev]-TGTGAAGCACTGATTCTTCTG |
| IN10-405HIBCHf  IN11+200HIBCHr | [-21M13]- TGTGAACTCCTCCTTGAGAG  [M13-Rev]-ACGTGAGATTGCACCACTGC |
| IN11-61HIBCHf  IN12+581HIBCHr | [-21M13]- AAGCCTATGTTCAGATTCATC  [M13-Rev]-CTCCATAGGACAAGGAGGAC |
| IN12-383HIBCHf  IN13+160HIBCHr | [-21M13]- GACCTATGCCGATTCAAGAC  [M13-Rev]-GGTAGCAATACTTCCACACATC |
| IN13-148HIBCHf  IN14+398HIBCHr | [-21M13]- TAGGAGGTGGTGATCAGAGAG  [M13-Rev]-AACAGTGGCCAAGTTATGTG |
| IN14-506HIBCHf  IN15+55HIBCHr | [-21M13]- AAATCCAGAGCTGTCTGAAC  [M13-Rev]-ACATGCTGTAGATTGCCAAC |

^1^All primers contain a -21M13 (5'-TGTAAAACGACGGCCAGT-3') or an M13-Rev (5'-CAGGAAACAGCTATGACC-3') extension.
